# Targeting EZH1/2: tackling epigenetic resistance advances cancer therapy

**DOI:** 10.1002/mco2.70022

**Published:** 2025-01-03

**Authors:** Ram Abou Zaki, Assam El‐Osta

**Affiliations:** ^1^ Epigenetics in Human Health and Disease Program Baker Heart and Diabetes Institute Melbourne Victoria Australia; ^2^ Baker Department of Cardiometabolic Health The University of Melbourne Parkville Australia; ^3^ Department of Medicine and Therapeutics The Chinese University of Hong Kong (CUHK) Hong Kong SAR China

1

In a recent paper published in *Nature*, the mechanisms of action and resistance in histone lysine methylation‐targeted treatment of adult T‐cell leukemia/lymphoma (ATL) were characterised.^1^ The study showed tumor regression begins as early as the first week of valemetostat treatment with patients enrolled in Phase I and II trials achieving complete remission. This is mediated by reducing histone methylation and activating the expression of tumor suppressor genes (TSG). This innovative study defined new mechanisms of cancer resistance highlighting the importance of epigenetic influences that could be targeted to improve drug efficacy.

ATL is a rare and highly aggressive hematological malignancy. A total of 1000 cases are diagnosed annually in Japan, because of the high prevalence of human T‐cell leukemia virus type 1. While the first clinical trial started in Japan, the hematological malignancy is not regional with a worldwide prevalence of 3000 cases. Indeed, according to the US Leukemia Foundation, treatment of ATL is subtype dependent. In practice, monitoring patients without treatment is applicable in smoldering ATL whereas the acute and lymphomatous subtypes require antiviral drugs and cytokine therapy combined with chemotherapy regimens.^2^ While therapeutic options differ among countries, treatment is limited and often involve multiple drugs with relapse in 90% of patients.^3^ The median survival for the acute form of ATL representing the most common subtype is 8 months. The aggressive nature of the malignancy combined with the high mortality rate has renewed interest to develop more targeted ATL therapies.

Valemetostat is the first‐in‐class EZH1/2 inhibitor for use in ATL. Competitively blocking the histone methyltransferase enzymes, enhancer of zeste homolog 2 (EZH2), and its highly related homolog EZH1, valemetostat received approval from the Japan Ministry of Health, Labour and Welfare in September 2022. EZH1/2 is the histone methyltransferase of polycomb repressive complex 2 (PRC2) including the corepressive complex containing embryonic ectoderm development (EED) subunit. The EZH1 and EZH2 enzymes write the trimethylation mark at lysine position 27 of histone H3 (H3K27me3) to regulate the formation of facultative heterochromatin for lineage commitment and cell identity (Figure 1A). Yamagishi and colleagues^1^ convincingly showed the therapeutic benefit of valemetostat on ATL regression was directly related to reduced H3K27me3 deposition on TSGs thereby activating their expression (Figure 1B).

**FIGURE 1:**
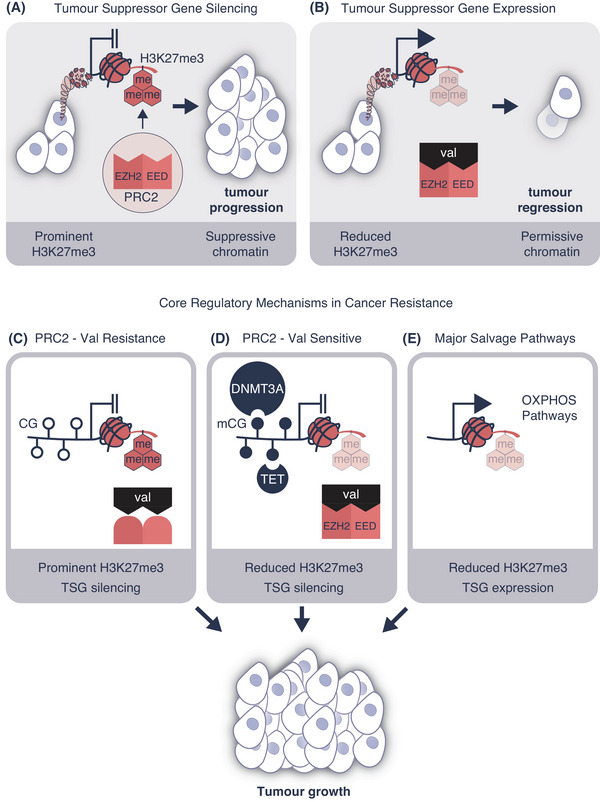
Mechanisms of action and resistance in histone methylation‐targeted therapy. (A) EZH2 and EED are subunits of the polycomb repressive complex 2 (PRC2) and together form valemetostat the binding pocket. PRC2 is responsible for writing H3K27me3, heterochromatin formation, and the silencing of tumor suppressor gene (TSG) expression implicated in tumor progression. (B) Valemetostat competitively binds the EED and EZH2 pockets to inhibit PRC2 methyltransferase activity causing precipitous reduction of H3K27me3 gene content and active TSG expression. This mechanism of action is responsible for valemetostat therapeutic benefit and tumor regression. (C) Three key mechanisms of valemetostat resistance were identified in ATL. PRC2–valemetostat resistance mediated by amino acid substitutions in EZH2 and EED subunits reduces the affinity of valemetostat binding to the PRC2 restoring H3K27me3 deposition and silencing TSG expression. This is likely to be independent of DNA methylation at CG sites. (D) PRC2–valemetostat sensitivity involves compensatory DNA hypermethylation achieved by reduced TET function or DNMT3A overexpression and elevating DNA methylation at TSGs. DNA methylation (mCG) dominates histone hypomethylation causing tumor growth. (E) Activation of major salvage pathways in ATL overcome valemetostat induced TSG expression. The genes that participate in these pathways regulate oxidative phosphorylation (OXPHOS). This mechanism of resistance drives tumor growth.

Demonstrating drug efficacy, the investigators subsequently tackled understanding the biology behind heterochromatin formation. Fundamental research of this type established resistance mechanisms that contribute to cancer relapse during drug therapy. The study showed almost 50% of ATL patients acquired somatic mutations in the valemetostat‐binding pocket of the PRC2. The study also identified important mutations that influence amino acid substitutions such as Y111S/Y111C/Y111H/Y111N/Y661N in EZH2 and H213R in the EED proteins (Figure 1C).

Genetic changes were also observed in ATL such as single nucleotide insertions, deletions, and copy number variations. Valemetostat inhibition of EZH1/2 reduced H3K27me3 causing compensatory DNA methyltransferase 3 alpha (DNMT3A) overexpression. The other major line of epigenetic resistance identified in the study was reduced TET2 function, this protein is a methylcytosine dioxygenase that catalyzes the conversion of methylcytosine to 5‐hydroxymethylcytosine. While TET2 activity did not alter H3K27me3 gene content, it was associated with the compensatory gain of methylated CG sites directly silencing TSG function (Figure 1D). The practical outcome of these studies was restored valemetostat efficacy following low dose decitabine treatment. This strategy was effective in overcoming ATL resistance despite DNMT3A overexpression and reduced TET2 function. While the precise mechanism of action remains poorly defined, we propose the combination of decitabine and valemetostat treatment could be superior to single agent therapy.

Two distinguishable ATL subclusters were also identified based on clinically meaningful valemetostat response. Subcluster‐A was more vulnerable to treatment whereas subcluster‐B developed valemetostat resistance involving major salvage pathways (Figure 1E). Previous Phase I and II trials have shown that not all ATL patients respond equivalently (NCT04703192, NCT041021150). While the regulatory reasons behind United States Food and Drug Administration denial are complicated,^4^ the variable patient response to valemetostat is now understandable. Two recent ATL trials at different study sites exemplify this clinical position. Trial NCT041021150 published in September 2022 evaluated valemetostat at 200 mg/day in 25 patients that had previously received three‐prior lines of therapy. The response rate to valemetostat was 48% and included five complete and seven partial patient remissions. Treatment‐emergent adverse events such as thrombocytopenia, anemia, and other cytopenias were manageable. That trial showed promising efficacy and tolerability of valemetostat in heavily pretreated patients. In contrast, an independent trial sharing a similar study design showed questionably modest response rates (May 2024, NCT04703192). Only two of the 22 patients with refractory ATL completed the study, with the remaining participants withdrawing because of disease progression or relapse. While these study findings are preliminary, the indication of variability in response to valemetostat is arguably attributed to core regulatory mechanisms in cancer resistance summarized in Figure 1C–E.

Histones do more than simply compress DNA and nowhere is this more evident than the H3K27me3 mark, which plays an important role in gene regulation and tumor progression. In the same way, the methylation mark H3K27me3 that is written by EZH1/2 is also subject to enzymatic erasure by histone demethylases. The removal of this methylation mark predictably influences chromatin decompaction. These regulatory events actively turn genes on, restoring cell growth and blocking cancer progression.^5^ Indeed, there is mounting evidence that drug resistance mechanisms in ATL are time dependent, with estimates indicating clinical presentation taking more than 2 months to develop. This discovery only emphasizes the need to conduct further tests on the prospective superiority of combination therapy. The study by Yamagishi and colleagues^1^ has also influenced subsequent clinical trials using valemetostat in combination with other drugs. For example, a recent Phase I/II study is focused on dose escalation of valemetostat in combination with atezolizumab and bevacizumab for the treatment of advanced hepatocellular carcinoma in patients that have not received prior therapy (NCT06294548). While that trial is ongoing and study outcomes pendent, this combinatorial strategy could confer synergic benefit. In yet another open label trial using valemetostat in combination with rituximab and lenalidomide for the treatment of relapsed follicular lymphoma, that study was designed to evaluate pharmacokinetics and safety. This Phase I/II trial is presently recruiting patients at M.D. Anderson Cancer Centre.

The important work has started. The ground under cancer treatment is shifting, and while this is not an earthquake of change, these clinical trials register the tremors that could influence cancer therapy. The outcomes for these important trials are pending study updates.

## AUTHOR CONTRIBUTIONS

Ram Abou Zaki and Assam El‐Osta contributed equally in writing the manuscript. Assam El‐Osta drew the figures. Both authors have read and approved the article.

## CONFLICT OF INTEREST STATEMENT

None declared.

## ETHICS STATEMENT

Not applicable.

## Data Availability

Not applicable.
